# Vasopressors and inotropes in cardiogenic shock patients: an analysis of the MIMIC-IV database

**DOI:** 10.3389/fcvm.2023.1300839

**Published:** 2023-11-29

**Authors:** Bryan Richard Sasmita, ChuanYing Wang, Siyuan Xie

**Affiliations:** ^1^ Department of Cardiology, The First Affiliated Hospital of Chongqing Medical University, Chongqing, China; ^2^ Department of Medical Administration, The Sixth People’s Hospital of Chongqing, Chongqing, China

**Keywords:** cardiogenic shock, vasopressors, inotropes, 30-day mortality, pharmacological support

## Abstract

**Introduction:**

Pharmacological support has become the mainstay therapy in patients with cardiogenic shock (CS). Unfortunately, the clinical benefits of such potent drugs remain unclear, therefore, the present study aims to elucidate the safety and efficacy of vasoactive agents in CS patients.

**Methods:**

Medical Information Mart for Intensive Care (MIMIC) IV databases were used for this retrospective study. The primary outcome of this study was 30-day all-cause mortality. The subgroup analysis of was the relationship between the combined use of vasopressors and inotropes and 30-day all-cause mortality.

**Results:**

A total of 2,216 patients diagnosed with CS were enrolled in this study. The non-survivors group was more likely to be older, presented with chronic kidney disease, have a lower systolic blood pressure, lower heart rate, and higher respiratory rate (all *p* < 0.05). In the multivariate Cox regression analysis, only dopamine [HR (95%CI): 1.219 (1.003–1.482)], norepinephrine [HR (95%CI): 2.528 (1.829–3.493)], and milrinone [HR (95%CI): 0.664 (0.512–0.861)] remained an independent predictor for 30-day all-cause mortality. Furthermore, a subgroup analysis was performed and found that no statistically significant difference between no vasopressor/inotrope use and 1 vasopressor/inotrope use (*p* = 0.107). Meanwhile, a substantial deterioration of cumulative survival was observed when a combination of 2 or more vasopressors/inotropes was used in CS patients in comparison with no vasopressor/inotrope or only 1 vasopressor/inotrope use (all *p* < 0.05).

**Conclusions:**

Using vasopressors/inotropes agents was associated with a higher risk of 30-day all-cause mortality in CS patients. In addition, only milrinone was associated with a better prognosis among the available vasoactive agents.

## 1. Introduction

Cardiogenic shock (CS) is a highly morbid clinical syndrome associated with inadequate tissue perfusion resulting from the heart's inability to function effectively. Acute decompensated heart failure and acute myocardial infarction are the most common causes of CS, making mechanical and pharmacological support as the mainstay therapy to improve short-term survival (1–3). Unfortunately, despite advances in treatment options, CS mortality remains as high as 50% (3, 4), thus posing a therapeutic challenge for clinicians.

The goal of CS treatment can be categorized into two types: first is to treat and identify the underlying cause; second is to provide adequate hemodynamic support through vasoactive medications. Vasopressors or inotropes have become crucial in hemodynamic management for most patients with CS due to their effect on reducing adrenergic stress (decatecholaminisation) (5). These medications are routinely used as a supportive therapy until definitive therapies can be provided. Despite its frequent use, vasopressors or inotropes may increase left ventricular afterload, causing higher myocardial oxygen demand, greater infarct size, and a higher risk of developing arrhythmia (6). In addition, limited evidence is available regarding vasopressors or inotropes' efficacy in improving clinical outcomes in CS patients; hence, the role of pharmacologic hemodynamic stabilization in CS remains inconclusive (4, 6, 7). This study aimed to elucidate the relationship between vasopressor or inotrope use and 30-day mortality in CS patients in real-world conditions using the Medical Information Mart for Intensive Care IV (MIMIC-IV) database.

## 2. Methods

### 2.1. Data source and ethics

The data for this study were obtained from the MIMIC-IV database (8, 9), which is a publicly accessible clinical database containing comprehensive information from the Beth Israel Deaconess Medical Centre in Boston, Massachusetts, USA. This database includes deidentified data from 76,540 intensive care unit stays between 2008 and 2019 at the Beth Israel Deaconess Medical Centre. It comprised of charted events, such as laboratory data, vital status, demographics, discharge summaries, and diagnostic information using the International Classification of Diseases, Ninth and Tenth Revision (ICD-9 and ICD-10). The establishment of the database was approved by the institutional review boards of the Massachusetts Institute of Technology (Cambridge, MA) and Beth Israel Deaconess Medical Centre (Boston, MA). Consent was obtained for the original data collection; therefore, the Institutional Research Committee of the Sixth People's Hospital of Chongqing (Chongqing, China) waived the informed consent required for the present study. The author B.R.S has finished the Collaborative Institutional Training Initiative Examination (Certification number: 57772730) and approved using this database. Furthermore, we understand and comply with relevant research ethics requirements and regulations regarding using the data in our study (Declaration of Helsinki).

### 2.2. Study population

The study population comprised adults with CS as defined by the ICD-9 codes (code = 785.51) and ICD-10 codes (code = R57.0). Patients younger than 16 years old, with incomplete information about study outcomes and treatment-related data, and staying less than 24 h in the ICU were excluded from this study.

### 2.3. Data extraction

Clinical data, laboratory data, demographic data, diagnostic information, and procedures were extracted using Structured Query Language (SQL). Demographic data included sex, age, body mass index (BMI), and smoking status. Laboratory and clinical data included vital signs, hospital death, comorbidities, lactate, cardiac troponin T, urea, creatinine, leukocyte, hemoglobin, neutrophil, lymphocyte, glucose, invasive and non-invasive treatments. The laboratory data were extracted within the first 24 h after ICU admission. Data use of vasopressors or inotropes were also extracted from the database. Vasopressors or inotropes used during ICU admission included dopamine, dobutamine, epinephrine, norepinephrine, and milrinone.

### 2.4. Outcomes

The primary endpoint was the association between vasopressors or inotropes use with 30-day all-cause mortality in CS patients. In addition, a subgroup analysis will be performed to assess the relationship between the combined use of vasopressors and inotropes and 30-day all-cause mortality in CS patients.

### 2.5. Statistical analysis

The data analysis was performed using SPSS version 25.0 (IBM, USA), MedCalc statistical software 19.2.6, and GraphPad Prism 8.4.3. For continuous variables with a normal distribution, the mean and standard deviation (SD) were reported, while for variables with a skewed distribution, the median and interquartile range (25% - 75%) were reported. Categorical variables were presented as the number of cases and percentages, and the Pearson test was used to compare differences between groups. The patients were then categorized into two groups based on their 30-day survival status, and the cumulative survival time of the cardiovascular system (CS) with each vasopressor or inotrope was analyzed using the Kaplan–Meier (K–M) method.

To analyze the independent relationship between vasopressors and inotropes and 30-day all-cause mortality, both univariate and multivariate Cox regression analyses were conducted. The multivariate Cox regression analysis was adjusted based on clinically relevant variables or variables that had *p*-values below 0.05 in the univariate analysis. The Cox proportional model was applied using a forward stepwise method, with an entry criteria of *p* < 0.05. The adjusted hazard ratio (HR) and its corresponding 95% confidence interval (CI) were calculated. Statistical significance was determined by a two-sided *p*-value < 0.05. If the HR was greater than 1.0 with a *p*-value < 0.05, it indicated that the parameter was a risk factor for 30-day mortality. Conversely, if the HR was less than 1.0 with a *p*-value < 0.05, it indicated that the parameter was a protective factor against 30-day mortality.

## 3. Results

**Figure 1:**
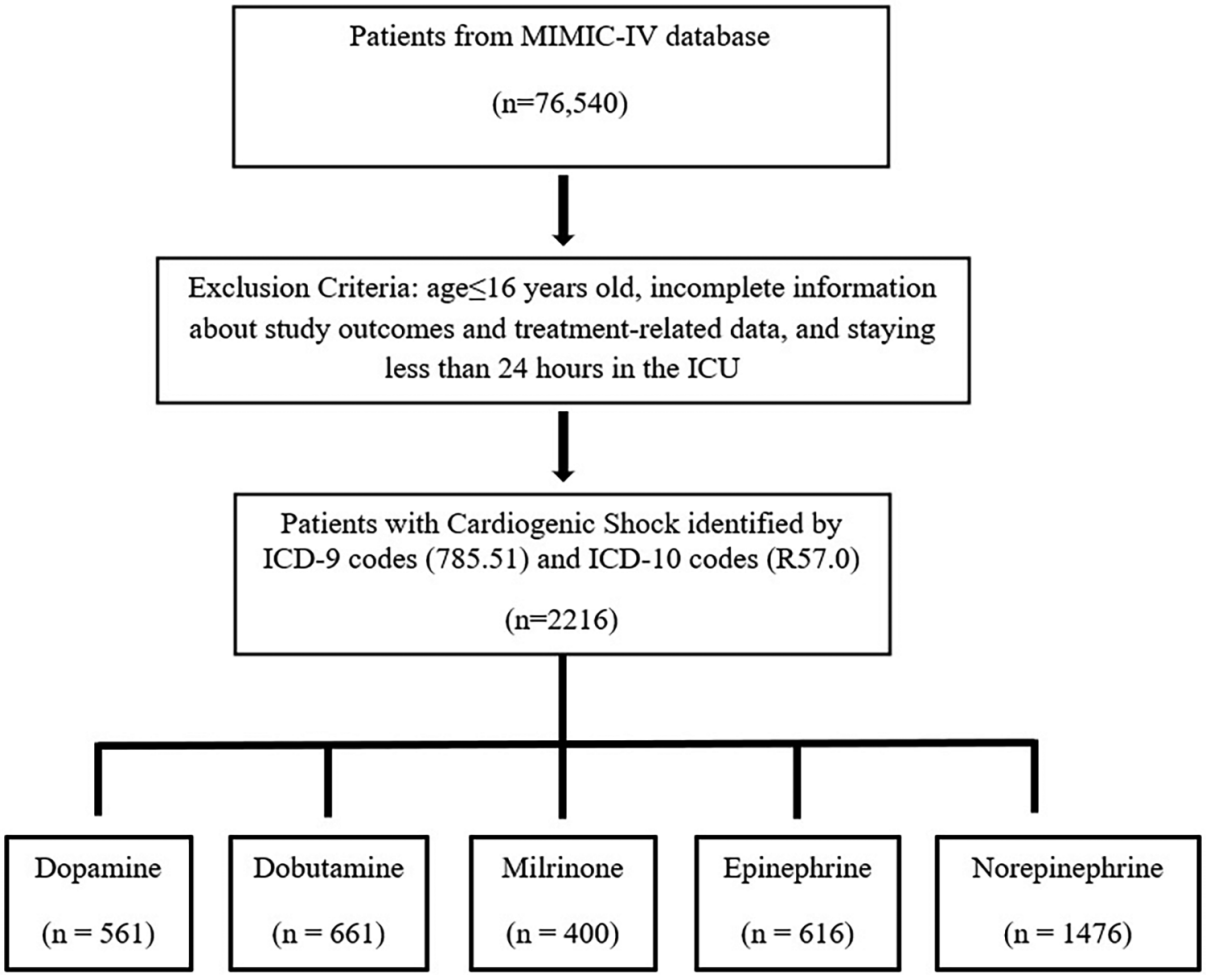
The flow chart of included patients.

In total, 2,216 patients diagnosed with CS met the inclusion criteria (Figure 1). During a 30-day follow-up period, a total of 922 (41.6%) patients died. The clinical characteristics between survivors and non-survivors were analyzed. As presented in Table 1, the baseline characteristics between the two groups and demonstrated that the non-survivors group was more likely to be older and presented with chronic kidney disease (CKD) (all *p* < 0.05). Furthermore, in terms of vital signs, the non-survivors presented with lower heart rate (HR), lower systolic blood pressure (SBP), lower diastolic blood pressure (DBP), and higher respiratory rate (RR) (all *p* < 0.05).

**Table 1:** Baseline demographics between survivors and non-survivors group.

Baseline characteristics	Survivors	Non-survivors	*p* value
	*N* = 1,294	*N* = 922	
Age (years)	64.86 ± 14.64	72.02 ± 13.62	<0.001
Male (%)	815 (63.0)	530 (57.5)	0.009
BMI (kg/m^2^)	28.46 ± 6.08	27.96 ± 6.52	0.124
Smoking (%)	361 (27.9)	206 (22.3)	0.813
Comorbidities (%)			
Primary pypertension	372 (28.7)	218 (23.6)	0.007
Type 2 diabetes mellitus	138 (10.7)	108 (11.7)	0.439
Acute myocardial infarction	286 (22.1)	208 (22.6)	0.799
Heart failure	987 (76.3)	596 (64.6)	<0.001
Atrial fibrillation	541 (41.8)	397 (43.1)	0.557
Hyperlipidemia	590 (45.6)	404 (43.8)	0.407
Cerebral infarction	62 (4.8)	50 (5.4)	0.504
COPD	97 (7.5)	71 (7.7)	0.858
Chronic kidney disease	369 (28.5)	341 (37.0)	<0.001
Dilated cardiomyopathy	56 (4.3)	12 (1.3)	<0.001
Ischemic cardiomyopathy	203 (15.7)	170 (18.4)	0.088
Vital signs			
Heart rate (bpm)	88.0 (76.0–105.0)	92.0 (78.0–109.0)	0.001
SBP (mmHg)	109.0 (95.5–123.0)	105.0 (91.0–119.0)	0.006
DBP (mmHg)	59.0 (49.0–69.0)	56.0 (47.0–67.0)	0.005
Respiratory rate	20.0 (16.0–24.0)	21.0 (17.0–25.0)	<0.001

^a^ BMI, body mass index; COPD, chronic obstructive pulmonary disease; BPM, beats/ minute; SBP, systolic blood pressure; DBP, diastolic blood pressure.

The laboratory parameters and treatment between survivors and non-survivors is showed in Table 2. In terms of laboratory parameters, the non-survivors group was more likely to have lower leukocyte and lymphocyte, higher lactate, higher urea, higher creatinine, and higher glucose level (all *p* < 0.05). Additionally, in terms of treatment, the non-survivors were less likely to receive milrinone, aspirin, beta-blockers, angiotensin-converting enzyme inhibitors (ACEI), PY2Y12 inhibitors, and angiotensin receptor blockers (ARBs); however, they had a higher usage of mechanical ventilation, hemodialysis treatment, dopamine, dobutamine, and norepinephrine (all *p* < 0.05).

**Table 2:** Comparison of laboratory parameters and treatments.

	Survivors	Non-survivors	*p*-value
	*N* = 1,294	*N* = 922	
Lactate (mmol/L)	2.0 (1.4–3.1)	3.0 (1.8–5.6)	<0.001
Cardiac Troponin *T* (ng/ml)	0.23 (0.03–1.21)	0.29 (0.06–1.47)	0.062
Urea (mg/dl)	29.0 (19.0–46.0)	39.0 (25.0–60.0)	<0.001
Creatinine (mg/dl)	1.40 (1.00–2.13)	1.80 (1.23–2.80)	<0.001
Leukocyte (x10^9^/L)	13.12 (8.30–15.73)	12.50 (9.13–17.60)	0.014
Hemoglobin (g/dl)	11.5 (9.7–13.2)	10.7 (8.9–12.1)	<0.001
Neutrophil (%)	82.0 (75.0–86.9)	83.15 (76.0–88.0)	0.987
Lymphocyte (%)	9.55 (6.00–15.40)	8.0 (4.7–12.7)	0.017
Glucose (mg/dl)	145.0 (113.0–197.0)	157.0 (116.0–233.0)	<0.001
Types of MI (*n*, %)			
STEMI	80 (6.2)	64 (6.9)	0.475
NSTEMI	119 (9.2)	103 (11.2)	0.127
Treatments (%)			
Aspirin	1,037 (80.1)	609 (66.1)	<0.001
P2Y12 inhibitors	422 (32.6)	262 (28.4)	0.035
Beta-blockers	1,001 (77.4)	406 (44.0)	<0.001
Statins	925 (71.5)	513 (55.6)	<0.001
ACEI	625 (48.3)	100 (10.8)	<0.001
ARBs	58 (4.5)	10 (1.1)	<0.001
Dopamine	290 (22.4)	271 (29.4)	<0.001
Dobutamine	359 (27.7)	302 (32.8)	0.011
Epinephrine	342 (26.4)	274 (29.7)	0.089
Norepinephrine	743 (57.4)	733 (79.5)	<0.001
Milrinone	287 (22.2)	113 (12.3)	<0.001
IABP	89 (6.9)	52 (5.6)	0.239
Mechanical ventilation	331 (25.6)	310 (33.6)	<0.001
ECMO	12 (0.9)	13 (1.4)	0.289
Hemodialysis	38 (2.9)	45 (4.9)	0.019

^a^ STEMI, ST-elevation myocardial infarction; NSTEMI, Non-ST segment elevation myocardial infarction; ACEI, angiotensin converting enzymes inhibitors; ARBs, angiotensin receptor blockers; IABP, intra-aortic balloon pump.

**Figure 2:**
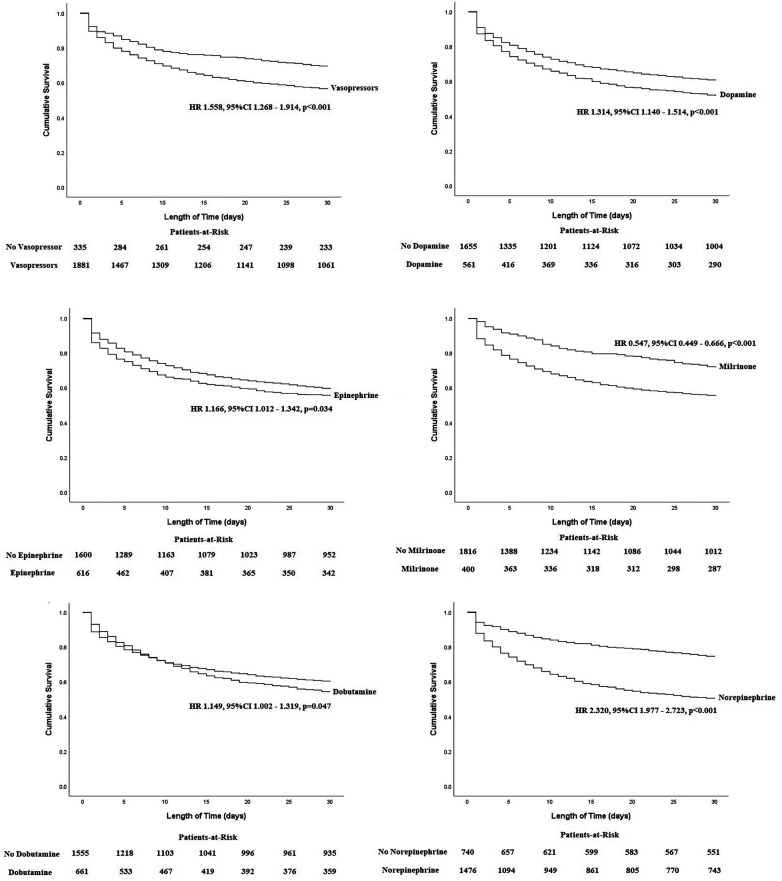
Kaplan–Meier curves between each vasopressor with 30-day all-cause mortality in patients with CS.

In Figure 2, the K-M survival curves illustrate the outcomes for patients treated with different vasopressors or inotropes over a 30-day period. It was found that the cumulative all-cause mortality was significantly higher among patients who received vasopressors or inotropes [HR (95%CI): 1.558 (1.268–1.914)], dopamine [HR (95%CI): 1.314 (1.140–1.514)], epinephrine [HR (95%CI): 1.166 (1.012–1.342)], dobutamine [HR (95%CI): 1.149 (1.002–1.319)], and norepinephrine [HR (95%CI): 2.320 (1.977–2.723)], while milrinone [HR (95%CI): 0.547 (0.449–0.666)] was the only inotrope that was associated with reduction of all-cause mortality. Furthermore, multivariate Cox regressions were conducted to determine the association between vasopressors or inotropes and the primary endpoint (Table 3). After multivariate adjustment, among all of the available vasopressors and inotropes, only dopamine [HR (95%CI): 1.219 (1.003–1.482)], norepinephrine [HR (95%CI): 2.528 (1.829–3.493)], and milrinone [HR (95%CI): 0.664 (0.512–0.861)] remained independent predictors of 30-day all-cause mortality. The other independent factors were age, SBP, HR, lactate, creatinine, cardiac arrest, and cardiac troponin (all *p* < 0.05).

**Table 3:** Multivariate Cox regression of primary endpoint.

	Univariable		Multivariable	
	HR (95% CI)	*p*	HR (95% CI)	*p*
Age (1 year increase)	1.028 (1.023–1.034)	<0.001	1.028 (1.020–1.035)	<0.001
Systolic blood pressure	0.995 (0.991–0.998)	0.003	0.996 (0.992–0.999)	0.017
Heart Rate	1.005 (1.002–1.008)	0.001	1.007 (1.002–1.011)	0.002
Acute myocardial infarction	1.048 (0.898–1.223)	0.552		
Acute respiratory failure	1.436 (1.261–1.634)	<0.001		
Chronic kidney disease	1.280 (1.120–1.463)	<0.001		
Cardiac arrest	1.950 (1.642–2.316)	<0.001	1.397 (1.115–1.750)	0.004
Lactate	1.138 (1.119–1.158)	<0.001	1.102 (1.077–1.127)	<0.001
Creatinine	1.099 (1.064–1.134)	<0.001	1.097 (1.045–1.152)	<0.001
Cardiac Troponin *T*	1.024 (1.007–1.041)	0.006	1.026 (1.005–1.047)	0.014
Dopamine	1.314 (1.140–1.514)	<0.001	1.219 (1.003–1.482)	0.047
Dobutamine	1.149 (1.002–1.319)	0.047		
Epinephrine	1.166 (1.012–1.342)	0.034		
Norepinephrine	2.320 (1.977–2.723)	<0.001	2.528 (1.829–3.493)	<0.001
Milrinone	0.547 (0.449–0.666)	<0.001	0.664 (0.512–0.861)	0.002

**Figure 3:**
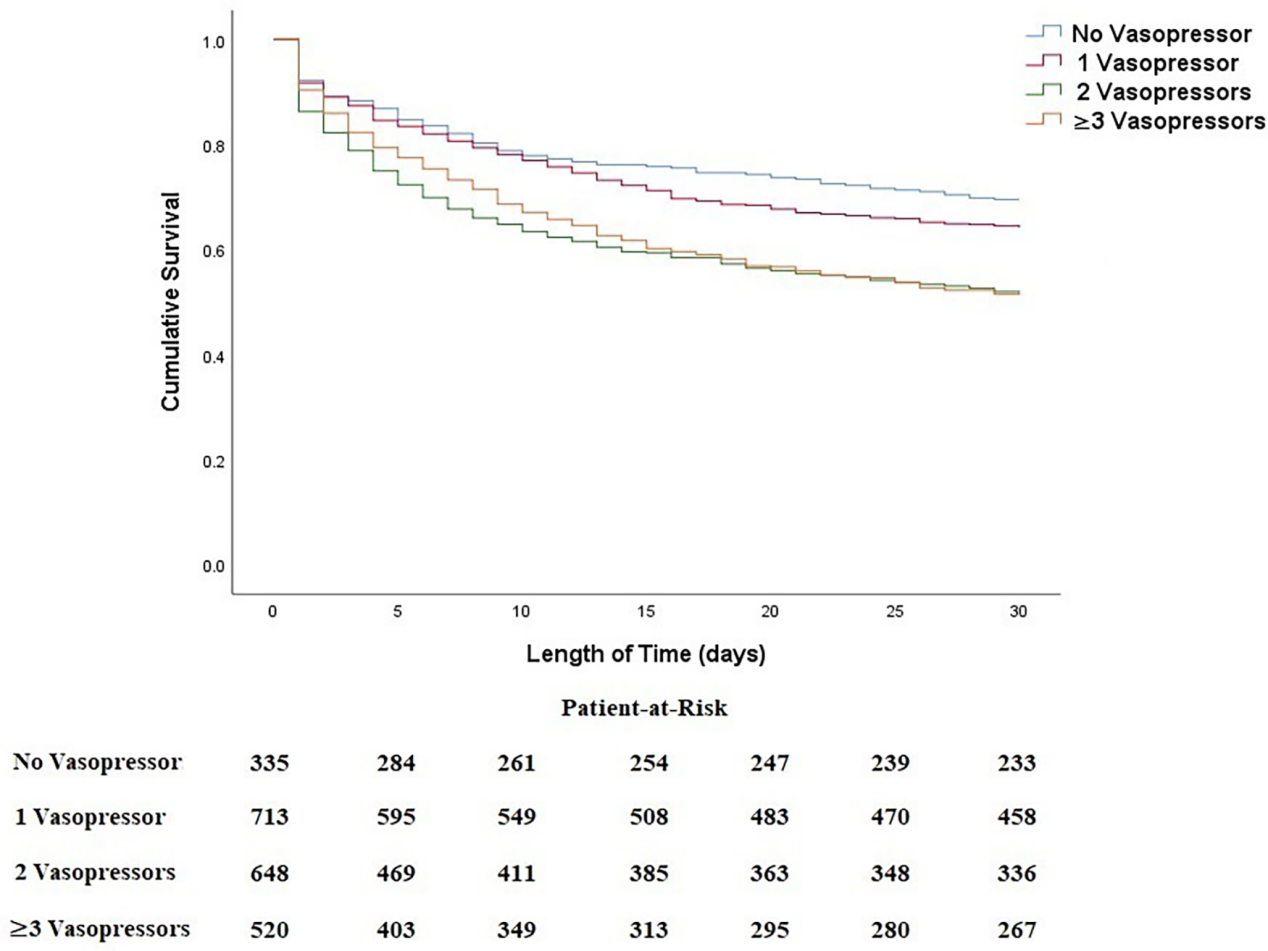
Kaplan–Meier curves between combined regimens of vasoactive agents with 30-day all-cause mortality in patients with CS.

A subgroup analysis was performed to provide a more detailed analysis of the relationship of the combination of vasopressors and inotropes with 30-day all-cause mortality (Figure 3 and Table 4). Results indicated no statistically significant difference between no vasopressor/inotrope use and 1 vasopressor/inotrope use (*p* = 0.107). On the other hand, there was a substantial deterioration of cumulative survival when a combination of 2 or 3 vasopressors/inotropes was used in CS patients in comparison with no vasopressor/inotrope or only 1 vasopressor/inotrope use (all *p* < 0.05). Furthermore, in order to provide a clearer relationship between each of the vasopressor or inotrope agents, we provided a more detailed analysis in patients with 1 vasopressor/inotrope use and compared each vasopressor or inotrope agent with one and another (Supplementary Figure S1). In the patients with 1 vasopressor/inotrope use, we found that 112 patients (5.1%) were given dopamine, 427 patients (19.3%) were given norepinephrine, 34 patients (1.5%) were given epinephrine, 33 patients (1.5%) were given milrinone, and 107 patients (4.8%) were given dobutamine. K-M curves were constructed to analyze their cumulative survival time, and we found that the cumulative all-cause mortality was significantly higher in patients with no vasopressor/inotrope use and in patients with norepinephrine use (Supplementary Figure S1). Moreover, in the Supplementary Figure S2, a higher risk of mortality was observed in patients with norepinephrine use (all log-rank *p* < 0.001).

**Table 4:** Log rank test outcome between combination of vasopressors with 30-day all-cause mortality.

Groups compared	Log rank test outcome 30-day mortality
No vasopressor vs. 1 vasopressor	0.107
No vasopressor vs. 2 vasopressors	<0.001
No vasopressor vs. ≥3 vasopressors	<0.001
1 vasopressor vs. 2 vasopressors	<0.001
1 vasopressor vs. ≥3 vasopressors	<0.001
2 vasopressors vs. ≥3 vasopressors	0.778

## 4. Discussion

The main findings from our study are as follows. First, vasopressors or inotropes are common in patients with CS. Second, among the available vasopressors or inotropes, epinephrine, dopamine, dobutamine, and norepinephrine were associated with a higher mortality risk, while milrinone was associated with a reduction of 30-day all-cause mortality. Third, only dopamine, norepinephrine, and milrinone remained independent predictors of 30-day all-cause mortality after multivariate adjustment. Fourth, in the subgroup analysis, we found that no vasopressor/inotrope or 1 vasopressor/inotrope use was not statistically significant, while a combination of 2 or more vasopressors and inotropes was associated with higher mortality risk.

CS is a lethal condition that requires immediate risk stratification and treatment. The development of CS has downstream effects on the entire circulation, causing tissue injury, inflammation, and hypoxia. It activates several compensatory mechanisms, such as endogenous sympathetic stimulation, peripheral vasoconstriction, heart rate, and myocardial contractility augmentation. Over time, persistent increases in cardiac afterload and a mismatch between myocardial oxygen supply and demand will eventually lead to a decompensated stage and cause a reduction of coronary perfusion (10). Therefore, a hemodynamic support strategy through vasoactive medications is essential to improve tissue perfusion and prevent the worsening of myocardial ischemia.

Vasopressors or inotropes are commonly administered in patients with CS, with a study found that approximately 90% of CS patients received vasoactive medications (11). Studies regarding the use of vasopressors or inotropes in CS patients are widely available; unfortunately, different vasopressors produce different outcomes. Thus, to date, there is no consensus about the benefit of vasoactive agents in improving the short-term prognosis of CS patients. A recent randomized controlled study demonstrated that epinephrine administration was more likely to develop refractory CS than norepinephrine in CS-complicating AMI patients (6). In addition, two meta-analyses suggested lower mortality with norepinephrine compared to other vasopressors (12, 13). Meanwhile, a recent retrospective cohort study by Lu et al. (14) used the MIMIC-III database to observe the association between 30-day mortality and norepinephrine use in CS patients. Lu and colleagues (14) found that norepinephrine use significantly increased short-term mortality. Furthermore, a randomized controlled study demonstrated that epinephrine administration was more likely to develop refractory CS than norepinephrine in CS-complicating AMI patients (6). Our finding is in agreement with the previous study (14), as after multivariate adjustments, we found that the 30-day all-cause mortality risk was the highest in norepinephrine use.

Previous studies have conducted comparisons between milrinone and dobutamine effectiveness and safety in CS patients, with most studies producing conflicting results. Mathew and colleagues (7) enrolled 192 patients with CS who were randomly assigned to receive either dobutamine or milrinone. In this randomized trial, they discovered that there was no significant distinction between milrinone and dobutamine in terms of the primary and secondary outcomes. Furthermore, a meta-analysis conducted by Biswas et al. (15) evaluated the difference between milrinone and dobutamine in acute decompensated heart failure (AHF) complicated CS, and the result showed a non-significant trend towards improved mortality in AHF-complicated CS. On the other hand, a recent study by Rodenas-Alesina E et al. (16) has demonstrated the benefit of milrinone compared with dobutamine in CS patients. This finding is in accordance with our study, as we found that administration of milrinone was associated with significant improvement in 30-day mortality. Several explanations are needed to explain these contradictory results. First, there is a significant difference in terms of study population and baseline characteristics between our study and others. It is noteworthy that in the present study, we conducted a retrospective cohort study in a general CS population, unlike Biswas et al. (15) and Levy et al. (6), who evaluate the efficacy of vasopressors in AHF-complicated CS and CS-complicating AMI, respectively. Second, each vasopressor agents have different mechanisms, for example, dobutamine and dopamine act on adrenergic and dopaminergic receptors, epinephrine and norepinephrine stimulates β- and α- adrenergic receptor, while milrinone prevents degradation of cyclic adenosine monophosphate through inhibition of phosphodiesterase-3 intracellular enzyme (17). Third, the proportion of vasopressors or inotropes used in each study differed; for example, in our research, norepinephrine was used in 66.6% of the patients, whereas a very low proportion of patients were on milrinone (18.1%), dopamine (25.3%), dobutamine (29.8%), and epinephrine (27.8%); thus, careful interpretation is needed.

In a more severe shock state, combinations of vasopressors and inotropes are commonly used; unfortunately, the optimal combination remains unknown. Moreover, little is known about the benefits and harmful effects of such combinations. Chen et al. (18) and Zhou et al. (19) performed a network meta-analysis regarding the combined use of vasopressors in septic shock patients and demonstrated the priority of combination regimens. Meanwhile, Rohm et al. (20) investigated the efficacy and safety of increased use of vasopressors or inotropes in CS patients who underwent an Impella device. Of 276 patients, Rohm and colleagues (20) found that mortality rates were higher as the usage of vasopressors and inotropes increased, with the most significant being observed in the use of 2–3 agents. The aforementioned findings differ from the present study, as in the subgroup analysis, we found a substantial deterioration of cumulative survival with the increased use of vasopressor/inotrope from 1–2. Moreover, a combination of 2 or 3 vasopressors and inotropes was associated with a higher risk of 30-day all-cause mortality compared with no or only one vasopressor/inotrope use. Different vasopressor combination regimens and population studies may be the best explanation for the disparity in results.

Our present study has several clinical implications. First, the use of single vasopressor/inotrope or combined vasopressors/inotropes is common in patients with CS; unfortunately, there is no consensus about the benefit or harm of such agents; hence, this study may provide some clarity. Second, based on the present study subgroup analysis, combining 2 or more vasopressors in CS patients was associated with a higher risk of mortality. Third, among available vasopressors or inotropes, only milrinone was associated with improvement of 30-day all-cause mortality, while norepinephrine use had the highest risk of 30-day mortality. Last but not least, the aforementioned clinical implications must be interpreted cautiously, as this was a hypothesis-generating retrospective study.

## 5. Limitations

Our research has several limitations that should be addressed. This retrospective study is based on the two public databases; thus, selection bias could not be excluded. Certain critical information or important laboratory parameters, such as left ventricular ejection fraction, causes of cardiogenic shock, central venous pressure, the decision of which vasopressors were used and discontinued, the diagnostic criteria of CS, and when to decide to use combined regimens of vasopressors, were not available. This study utilized MIMIC-IV, a large, retrospective, and single-center database to assess the relationship between vasopressor use and 30-day all-cause mortality. Unfortunately, this is a retrospective cohort study, thus, the generalization of conclusions should be interpreted cautiously. More studies with large samples should be conducted further to clarify vasopressors' efficacy and safety in CS patients.

## 6. Conclusions

Using vasopressors or inotropes agents was associated with a higher risk of 30-day all-cause mortality in CS patients. In addition, only milrinone was associated with a better prognosis among the available vasoactive agents.

## Data Availability

The original contributions presented in the study are included in the article/**Supplementary Material**, further inquiries can be directed to the corresponding author.

## Supplementary material

Supplement Figure S1 A comparison of each vasopressor use in 1 vasopressor/inotrope use group.

Supplement Figure S2 A comparison of each vasopressor or inotrope with another.
