# Supplementary figures and images for: Vasopressors and inotropes in cardiogenic shock patients: an analysis of the MIMIC-IV database
Source: Front Cardiovasc Med. 2023 Nov 29;10:1300839. doi: 10.3389/fcvm.2023.1300839 (PMC10716269; doi:10.3389/fcvm.2023.1300839)

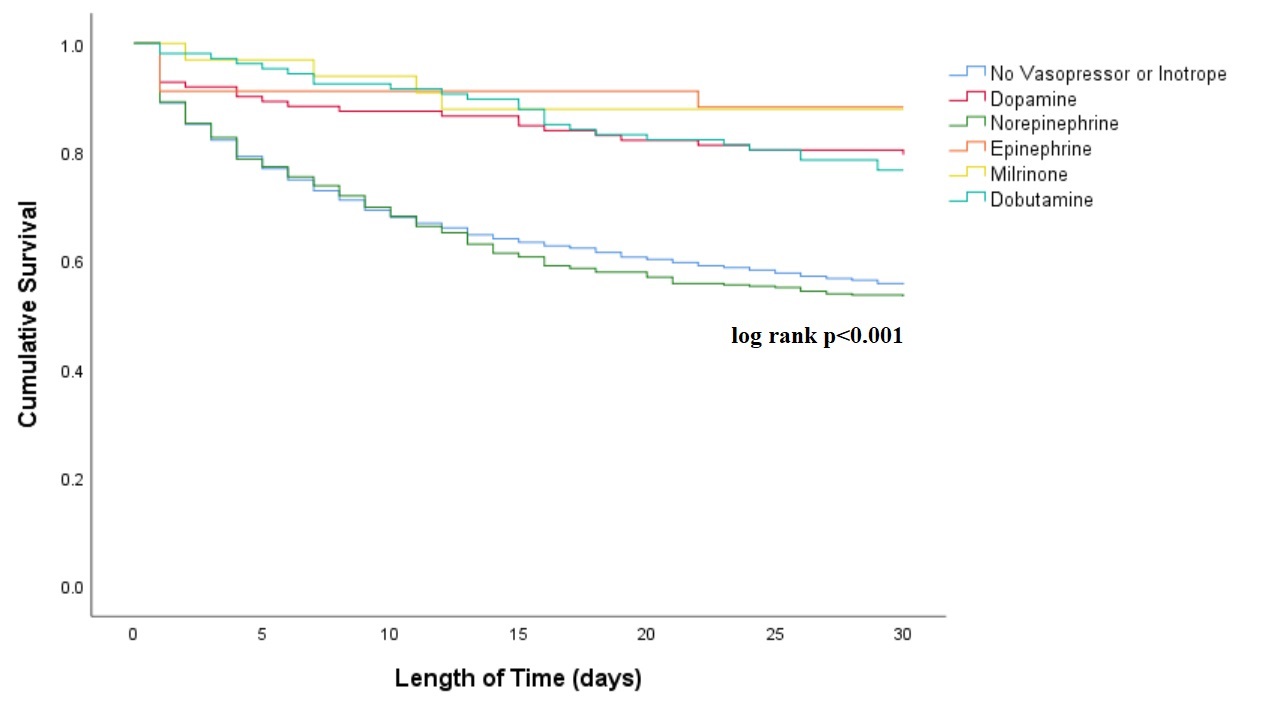

Supplement: Supplement Figure S1 — A comparison of each vasopressor use in 1 vasopressor/inotrope use group. [file Image1.jpeg]

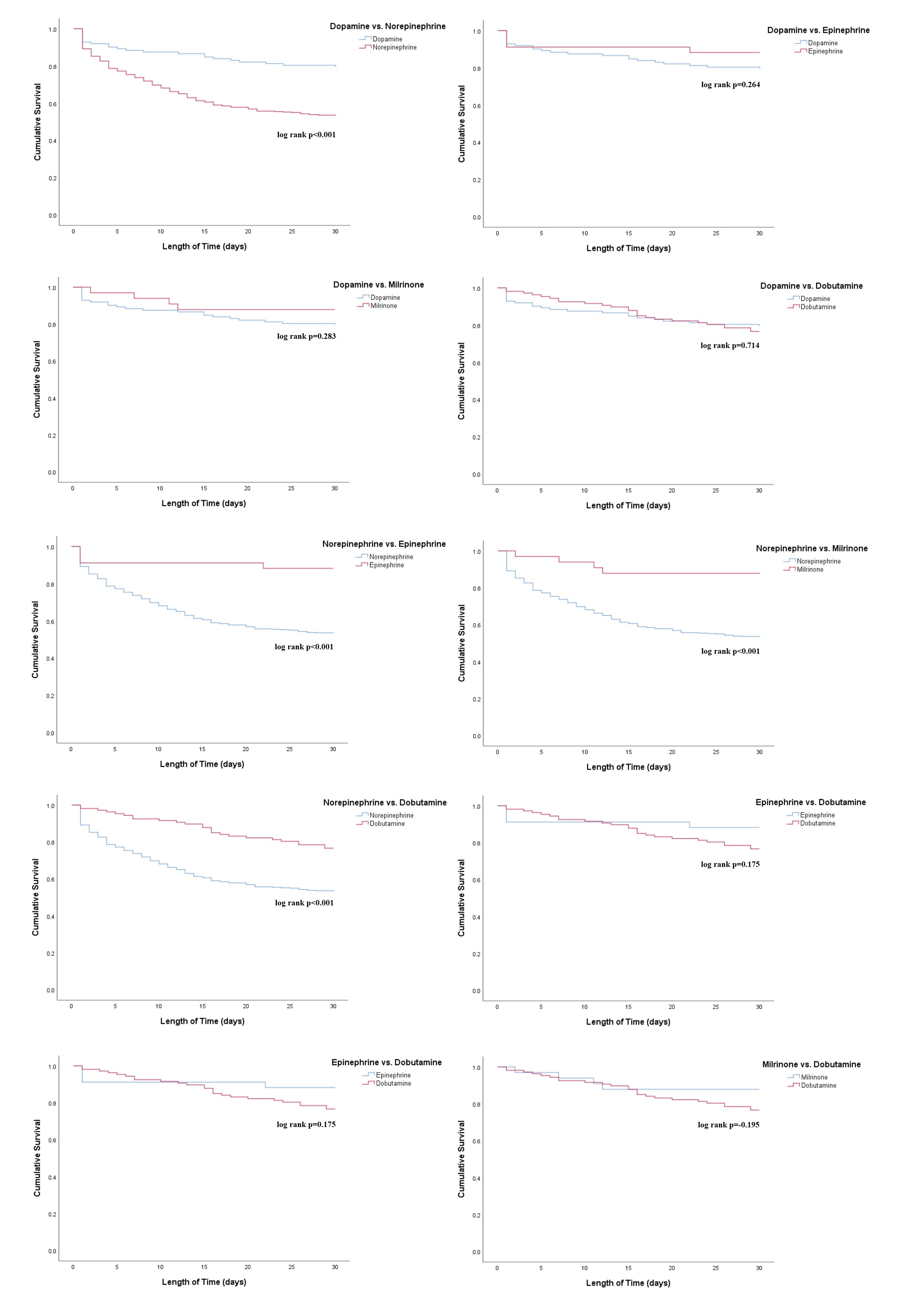

Supplement: Supplement Figure S2 — A comparison of each vasopressor or inotrope with another. [file Image2.jpeg]
